# Changes in brown adipose tissue induced by resveratrol and its analogue pterostilbene in rats fed with a high-fat high-fructose diet

**DOI:** 10.1007/s13105-023-00985-x

**Published:** 2023-10-16

**Authors:** Iker Gómez-García, Alfredo Fernández-Quintela, María Puy Portillo, Jenifer Trepiana

**Affiliations:** 1 Nutrition and Obesity Group, Department of Nutrition and Food Science, University of the Basque Country (UPV/EHU) and Lucio Lascaray Research Institute, Vitoria-Gasteiz, Spain; 2 Bioaraba Health Research Institute, Vitoria-Gasteiz, Spain; 3 CIBERobn Physiopathology of Obesity and Nutrition, Institute of Health Carlos III, Madrid, Spain

**Keywords:** Brown adipose tissue, Phenolic compounds, Pterostilbene, Resveratrol, Thermogenesis

## Abstract

Natural bioactive compounds have attracted a great deal of attention since some of them can act as thermogenesis activators. In recent years, special interest has been placed on resveratrol and its analogue pterostilbene, a dimethylether derivative that shows higher bioavailability. The aim of the present study is to compare the effects of resveratrol and its derivative pterostilbene on the thermogenic capacity of interscapular brown adipose tissue (iBAT) in rats under a high-fat high-fructose diet. Rats were divided into four experimental groups: control, high-fat high-fructose diet (HFHF) and HFHF diet supplemented with 30 mg/kg body weight/day of pterostilbene (PT30) or resveratrol (RSV30), for eight weeks. Weights of adipose tissues, iBAT triglycerides, carnitine palmitoyltransferase 1A (CPT1A) and citrate synthase (CS) activities, protein levels of uncoupling protein 1 (UCP1), sirtuins (SIRT1 and 3), AMP-activated protein kinase (AMPK), glucose transporter (GLUT4), fatty acid synthase (FAS), nuclear respiratory factor (NRF1), hormone-sensitive lipase (HSL), adipose triglyceride lipase (ATGL), CD36 and FATP1 fatty acid transporters, peroxisome proliferator-activated receptor gamma coactivator 1 (PGC1) activation and the batokines EPDR1 and NRG4 were assessed in iBAT. The results show that some key proteins related to thermogenesis were modified by either pterostilbene or resveratrol, although the lack of effects on other crucial proteins of the thermogenic machinery suggest that these compounds were not able to stimulate this process in iBAT. Overall, these data suggest that the effects of stilbenes on brown adipose tissue thermogenic capacity depend on the metabolic status, and more precisely on the presence or absence of obesity, although further studies are needed to confirm this hypothesis.

## Introduction

The non-shivering thermogenesis is a process characterised by the dissipation of energy as heat through mitochondrial respiration in response to cold or diet, due to the uncoupling of the oxidative phosphorylation [10]. This phenomenon is mediated by the action of the uncoupling protein 1 (UCP1), which is located in the inner mitochondrial membrane. Therefore, this protein dissipates the proton gradient across the membrane, producing heat instead of synthetising adenosine triphosphate (ATP) [8]. Brown adipose tissue (BAT) has been identified as the main thermogenic tissue [5]. BAT was believed to be found in small mammals and new-borns only, but nowadays it is well known that BAT depots are also present in adult humans, which may open the door to considering it as a possible target for body weight management [29].

In this regard, BAT triglyceride levels are reduced by thermogenic activity with the aim to produce heat. Thus, the lipids accumulated in this tissue are firstly oxidised and thereafter, circulating lipids are used as substrates [43]. In addition, in order to continue with this energy-demanding process, BAT also uses glucose from circulation, thus, regulating glucose homeostasis [46]. In this process, several proteins, such as PGC-1α and UCP1 play a key role; thus PGC-1α is highly expressed in BAT and through UCP1 up-regulation, it can promote the activation of brown adipocytes [9, 39]. In the same way, both AMPK through its phosphorylation, and SIRT1 through its acetylation can act as modulators of PGC-1α, allowing brown adipocytes taking up more glucose and burning more fat through non-shivering thermogenesis process [4, 13]. In addition, PGC-1α has also been defined as a promoter of increased mitochondrial number and mass, since it is along with NRF1 involved in enhancing expression of some key genes responsible for mitochondrial respiration and biogenesis [28, 53], like citrate synthase [40]. On the other hand, it is also known that SIRT3 has the ability to activate mitochondrial function and therefore plays an important role in the activation of BAT thermogenic machinery [51].Bearing this in mind, BAT has been considered in recent years as an important tissue with a protective role against some metabolic diseases, such as obesity and diabetes [5].

Diet is one of the crucial regulators of thermogenesis, due to the fact that energy intake and macronutrient composition have been related to thermogenesis modulation [41]. In this line, it has been reported that high dietary protein or carbohydrate intake is associated with diet-induced thermogenesis [19, 41]. Moreover, natural bioactive compounds have attracted a great deal of attention for the reason that some of them can act as thermogenesis activators in rodent models [3, 24, 34]. In recent years, special interest has been placed on resveratrol (3,5,4’-trihydroxy-trans-stilbene), a phenolic compound belonging to the stilbene group [14, 47]. Several studies have pointed out that this compound is a potential inducer of thermogenesis in rodent models [3, 34]. On the other hand, pterostilbene (4-[(*E*)-2-(3,5-dimethoxyphenyl)ethenyl]phenol), a dimethyl-ester of resveratrol, is also a potential tool for body weight management due to its role in the activation of thermogenesis [1, 24].

In this context, the present study aims to compare the effects of resveratrol and its derivative pterostilbene on the thermogenic capacity of BAT in rats under a high-fat high-fructose diet. The hypothesis is that indeed these phenolic compounds will contribute to increase the thermogenic capacity of BAT. Moreover, the mechanisms of action underlying the biological effects of each molecule are also studied. To the best of our knowledge, this is the first time that the efficacy of both phenolic compounds, in terms of thermogenesis, has been compared under the same experimental conditions.

## Material and methods

### Reagents

5,5’dithiobis (2-nitrobenzoic acid), acetyl CoA, DL-dithiothreitol (DTT), iodoacetamide, oxaloacetic acid, palmitoyl CoA, phenylmethylsulphonyl fluoride (PMSF) and triethanolamine hydrochloride were all purchased from Sigma-Aldrich (St Louis, MO, USA). Primary antibodies anti-SIRT1, -PGC-1α, -NRF1, -FAS and -CD36 were obtained from Abcam (Cambridge, UK). Primary antibodies anti-FATP1 (SLC27A1), -NRG4 and -EPDR1 were purchased from Thermo Fisher Scientific (Waltham, MA, USA). Primary antibodies anti-p-AMPK (Thr 172), -total AMPK, -Acetylated lysine, -pHSL (Ser 660), -total HSL, -α-tubulin and -histone H3 were obtained from Cell Signalling Technology (Danvers, MA, USA). Primary antibodies anti-UCP1, -SIRT3, -GLUT4 and -ATGL were purchased from Santa Cruz Biotechnology (Dallas, TX, USA).

### Animals, diets and experimental design

The study was performed using 40 male Wistar Rats (six-week-old, 140–150 g), purchased from Envigo (Barcelona, Spain). The animals were housed in pairs in polycarbonate metabolic cages, placed in a controlled air-conditioned room (22 ± 2 °C) and subjected to a 12-h light/dark cycle. After a 6-day adaptation period, animals were randomly distributed into four experimental groups of ten animals each: the control group (C) was fed a commercial standard diet (AIN-93G, OpenSource Diets, Gentofte, Denmark, D10012G); rats in the HFHF group received a high-fat-high-fructose diet containing 40% of lipids and 22% of fructose (OpenSource Diets, Gentofte, Denmark, D09100301); rats in the PT30 group were fed with the same high-fat-high-fructose diet enhanced with 30 mg pterostilbene/kg body weight/day; and rodents in the RSV30 group received the high-fat-high-fructose diet supplemented with 30 mg resveratrol/kg body weight/day. Both phenolic compounds were incorporated into the powdered diets on a daily basis, as previously reported [31]. Pterostilbene was kindly supplied by ChromaDex Inc. (Irvine, CA, USA) and resveratrol by Monteloeder (Elche, Alicante, Spain). All animals had free access to food and water. Food intake and body weight were measured daily.

After eight weeks under these experimental conditions, the animals were euthanised under anaesthesia (chloral hydrate) and sacrificed by cardiac exsanguination after 12 h of fasting. Interscapular BATs (iBAT) and white adipose tissue from different anatomical locations (epididymal, perirenal, mesenteric and subcutaneous from hindquarters) were dissected, weighed and immediately frozen in liquid nitrogen. All samples were stored at − 80 °C until analysis. All the experiments were performed in agreement with the Ethical Committee of the University of the Basque Country (document reference M20_2015_245 CUEID), following the European regulations (European Convention-Strasburg 1986, Directive 2003/65/EC and Recommendation 2007/526/EC).

### Triglyceride content

After homogenisation of iBAT samples, triglyceride levels were measured using a commercially available spectrophotometric kit (Spinreact, Girona, Spain). Triglyceride content values were expressed as mg triglycerides/mg tissue.

### Total and nuclear protein extraction and protein expression analysis by immunoblotting

For uncoupling protein 1 (UCP1), AMP-activated protein kinase (AMPK), sirtuin 1 (SIRT1), adipose triglyceride lipase (ATGL), hormone-sensitive lipase (HSL), fatty acid transport protein 1 (FATP1), cluster of differentiation 36 (CD36), glucose transporter type 4 (GLUT4), fatty acid synthase (FAS), sirtuin 3 (SIRT3), neuregulin 4 (NRG4), ependymin-related 1 (EPDR1) and α-tubulin total protein extraction was carried out. Therefore, 100 mg of iBAT was homogenised in 500µL of cellular PBS buffer (pH 7.4) with protease inhibitors. Homogenisation was performed by 5-s burst and 60% amplitude sonication with Branson SFX550 Sonifier (Saint Louis, MO, USA) fitted with a microtip and then centrifuged (800 g, 5 min, 4 °C). In the case of peroxisome proliferator-activated receptor gamma coactivator 1α (PGC-1α), nuclear respiratory factor 1 (NRF1) and histone H3, a nuclear protein extraction was carried out with 100 mg of iBAT, homogenised in 500µL of cellular PBS buffer (pH 7.4) with protease inhibitors, using Bullet Blender Storm 5 (Troy, NY, USA) with Zirconium oxide 2 mm beads during 10-m. Total and nuclear protein was spectrophotometrically quantified at 595 nm by Bradford assay [7] using bovine serum albumin (BSA) as standard.

Afterwards, immunoblot analyses were performed using 60 μg of total or nuclear protein from iBAT extracts, denaturalised for 5 min at 95 °C in Laemmli buffer [25] and separated by SDS-PAGE electrophoresis in 4–15% precast gels. Extracts were transferred onto PVDF membranes by electroblotting with constant amperage (1 mA/cm^2^). Next, the membranes were blocked with 4% BSA in PBS-Tween buffer for 2 h at room temperature. Subsequently, they were blotted overnight at 4 °C with the appropriate primary antibodies: UCP1 (1:1000), p-AMPK (Thr 172)(1:1000), AMPK (1:1000); SIRT1 (1:1000), PGC-1α (1:1000), Acetylated Lysine (1:1000), ATGL (1:1000), p-HSL (Ser 660) (1:1000), HSL (1:1000), FATP1 (SLC27A1) (1:1000), CD36 (1:1000), GLUT4 (1:1000), FAS (1:1000), NRF1 (1:1000), SIRT3 (1:1000), NRG4 (1:1000), EPDR1 (1:1000), histone H3 (1:1000) and α-tubulin (1:1000). Membranes were then incubated with the secondary horseradish peroxidase-conjugated antibody. Proteins were detected by the Forte Western HRP substrate (Millipore; Burlington, MA, USA), and the blots were imaged by scanning with the ChemiDoc™ MP Imaging System (Bio-Rad; Hercules, CA, USA). α-tubulin was used as housekeeping in total proteins and histone H3 in nuclear proteins.

### Enzyme activities

Carnitine palmitoyltransferase-1A (CPT-1A) activity was measured spectrophotometrically in the mitochondrial fraction as previously described [35]. The activity was expressed as nanomoles of coenzyme A formed per minute per milligram of protein. Citrate synthase (CS) activity was assessed spectrophotometrically following the Srere method [45], by measuring the appearance of free CoA. The specific activity was expressed as the amount of enzyme that transforms one nmol of substrate per min per mg protein.

### Statistical analysis

Results are presented as mean ± standard error of the means (SEM). Statistical analysis was performed using SPSS 25.0 (SPSS, Chicago, IL, USA). The normal distribution of the data was analysed by Shapiro–Wilks test. Data were analysed by one-way ANOVA followed by Newman–Keuls *post-hoc* test or by Kruskal–Wallis test as appropriate. Statistical significance was assessed at the *P* < 0.05 level. Effect size of the treatment was determined by Cohen’s *d*.

## Results

### Food and energy intake, total body weight, white adipose index and interscapular brown adipose tissue weight

As expected, no changes in food intake were found among groups. However, since the HFHF diet provided a higher amount of energy compared to the control, at the end of the experimental period, rats fed with the obesogenic diet (HFHF, PT30 and RSV30 groups) showed significantly greater energy intake (*p* < 0.05). Total body weight increased significantly in the HFHF group compared to rats fed with a standard diet (*p* < 0.05). The administration of resveratrol (30 mg/kg/d) for eight weeks prevented body weight increase (*p* < 0.05), although this effect was not observed in the rats supplemented with pterostilbene (30 mg/kg/d). Nevertheless, no changes among groups were observed in the white adipose index, calculated by the sum of different adipose depots (hindquarters subcutaneous, epididymal, perirenal and mesenteric). With regard to iBAT, the weight remained unchanged among groups (Table 1).

**Table 1:** Food and energy intake, final body weight, white adipose index, iBAT weight and epidydimal, perirenal, mesenteric and subcutaneous adipose tissue weight of rats fed with a control diet (C group), a high-fat high-fructose diet (HFHF group), a high-fat high-fructose diet supplemented with pterostilbene at a 30 mg/kg body weight/d dose (PT30 group) or a high-fat high-fructose diet supplemented with resveratrol at a 30 mg/kg body weight/d dose (RSV30 group) for eight weeks

	C	HFHF	PT30	RSV30	ANOVA
Food intake (g/day)	21 ± 0,2 a	21 ± 0,5 a	21 ± 0,3 a	19 ± 0,6 a	NS
Energy intake (kcal/day)	81,2 ± 0,9 b	93,3 ± 2,1 a	92,1 ± 1,6 a	86,2 ± 2,9 ab	*p* < 0.01
Final body weight (g)	393 ± 9 b	436 ± 10 a	428 ± 12 a	404 ± 15 ab	*p* < 0.05
Adipose index (%)	7,3 ± 0,6 a	8,0 ± 0,6 a	8,1 ± 0,5 a	7,7 ± 0,7 a	NS
iBAT (g)	0,76 ± 0,04 a	0,74 ± 0,06 a	0,82 ± 0,05 a	0,80 ± 0,04 a	NS
Epi + Peri + Mes + SC adipose tissue weight (g)	28.41 ± 1.97 a	35.01 ± 3.00 a	34.93 ± 2.83 a	31.30 ± 3.29 a	NS

*Epi* epidydimal; *iBAT* interscapular Brown Adipose Tissue; *Mes* mesenteric; *NS* not significant; *SC* subcutaneous; *Peri* perirenal. The adipose index is calculated as the ratio between the white adipose tissue weights (Epi + Peri + Mes + SC) and the final body weight. Values are presented as mean ± SEM. Values in the same row showing a different letter are significantly different (*p* < 0.05). Regarding the *post-hoc* test, Energy intake statistical differences were as follows: C *vs* HFHF *p* = 0.000, C *vs* PT30 *p* = 0.000; Final body weight statistical differences: C *vs* HFHF *p* = 0.004, C *vs* PT30 *p* = 0.03

### iBAT triglyceride content and protein expression of thermogenic capacity markers

**Fig. 1:**
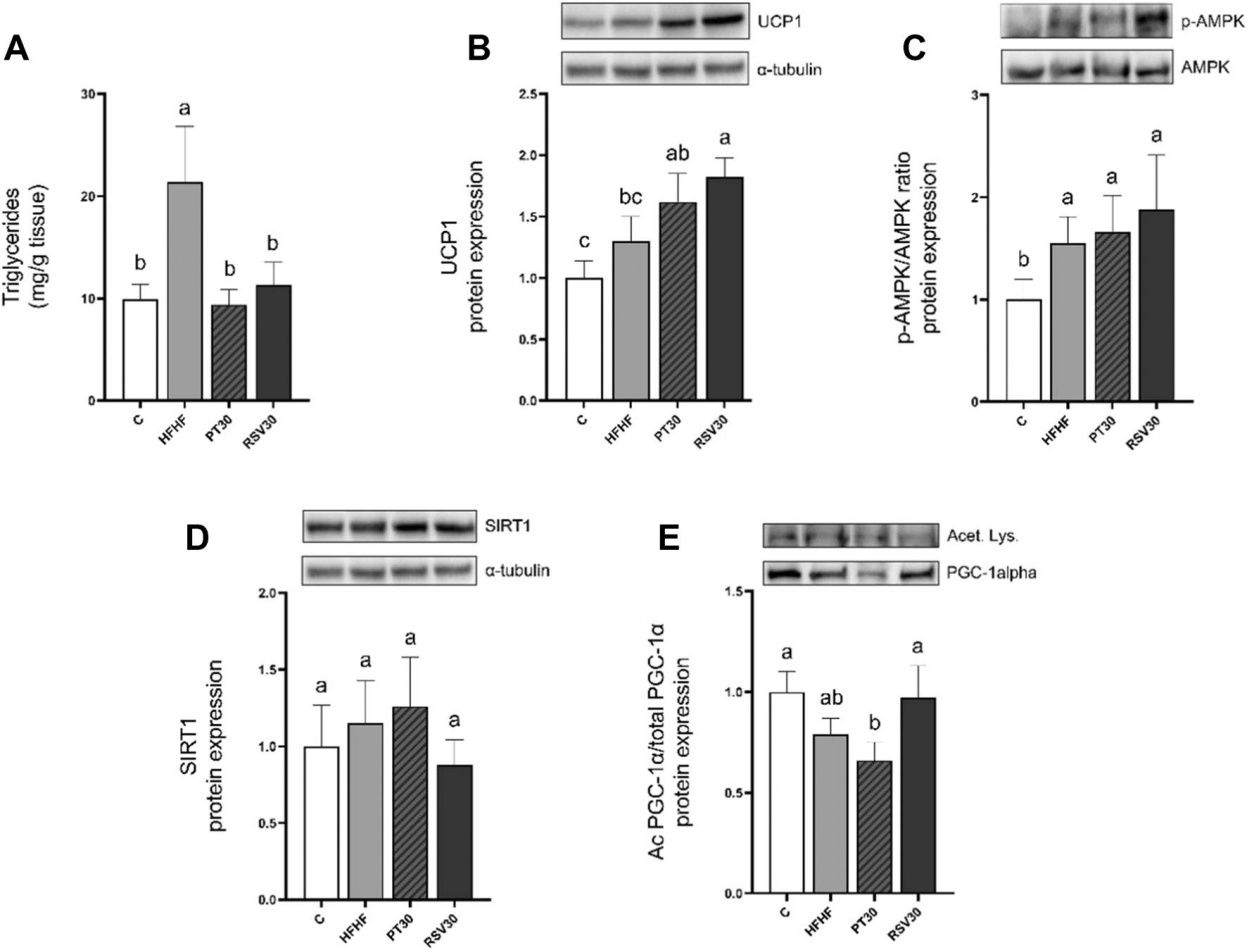
Triglyceride content (**A**) protein expression of UCP1 (**B**), p-AMPK/AMPK ratio (**C**) and protein expression of SIRT1 (**D**) and PGC-1α (**E**) in iBAT from rats fed with a control diet (C group), a high-fat high-fructose diet (HFHF group), a high-fat high-fructose diet supplemented with pterostilbene at a 30 mg/kg body weight/d dose (PT30 group) or a high-fat high-fructose diet supplemented with resveratrol at a 30 mg/kg body weight/d dose (RSV30 group) for eight weeks. Values are presented as mean ± SEM. Bars not sharing a common letter are significantly different (*p* < 0.05). AMPK: AMP-activated protein kinase; p-AMPK: phosphorylated AMP-activated protein kinase; PGC-1α: peroxisome proliferator-activated receptor gamma coactivator 1-α; SIRT1: sirtuin 1; TG: triglycerides; UCP1: uncoupling protein 1

As far as iBAT triglyceride accumulation is concerned, the HFHF diet caused a significant raise compared to the C group, and both phenolic compounds significantly prevented this increase (*p* < 0.05) (Fig. 1A). In order to analyse the effects induced by both pterostilbene and resveratrol on iBAT thermogenic capacity, the expression of UCP1 was measured (Fig. 1B). Although HFHF feeding did not modify UCP1 protein expression significantly (+ 29.9; *p* = 0.1), the treatment showed a large effect size (Cohen’s *d* =  + 1,8). Further, both phenolic compounds (PT30 and RSV30) induced a significant boost when compared to the control group (C) (*p* < 0.05 and *p* < 0.001, respectively). In comparison with the HFHF group, RSV30 showed increased protein expression (*p* < 0.05), whereas in PT30, only a tendency towards higher values (+ 32%; *p* = 0.1) was observed. Regarding effect size in comparison with HFHF, both PT30 and RSV30 produced a large treatment effect (Cohen’s *d* =  + 1.9 and + 2,9, respectively).

To better characterise the effects of both phenolic compounds on thermogenic capacity, AMPK, PGC-1α and SIRT1 were studied. Thus, the phosphorylation ratio of threonine 172 residue in AMPK protein was analysed to determine its activation status. When compared to the control group, HFHF showed increased protein expression (+ 55.7%; *p* < 0.05) and both phenolic compound-treated groups (PT30 and RSV30) displayed even higher values (*p* < 0.05). The differences between HFHF and phenolic compound-treated groups did not reach statistical significance (Fig. 1C). Regarding SIRT1 protein expression, no significant changes were observed among groups (Fig. 1D). As far as PGC-1α is concerned, decreased PGC-1α acetylation, which means a greater activation of this protein, no significant changes were found between the phenolic compounds and the HFHF group. Nevertheless, in the case of the PT30, a tendency towards lower acetylation values (-22.4%; *p* < 0.09) was observed (Fig. 1E).

**Fig. 2:**
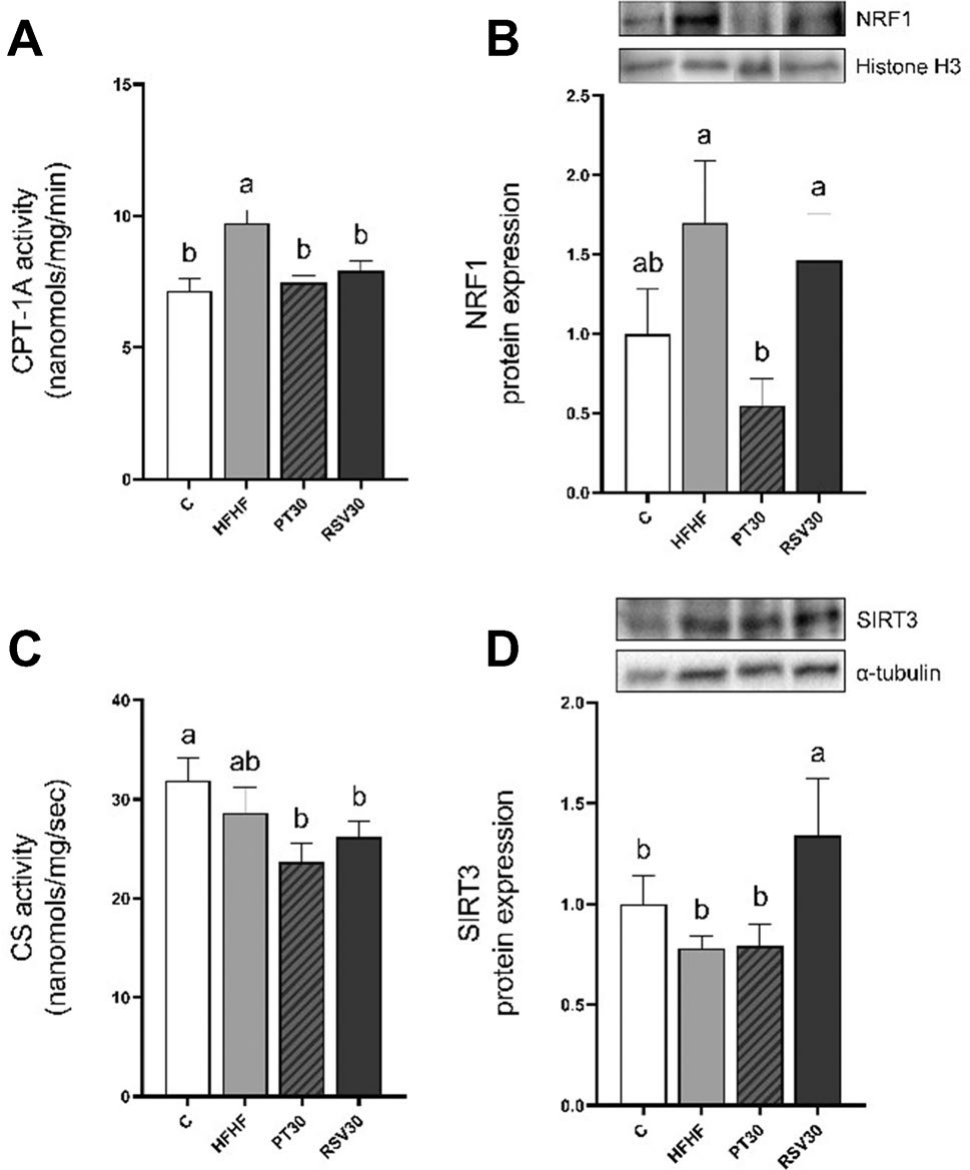
Enzymatic activity of CPT-1A (**A**) and CS (**C**) and protein expression of NRF1 (**B**) and SIRT3 (**D**) in iBAT from rats fed with a control diet (C group), a high-fat high-fructose diet (HFHF group), a high-fat high-fructose diet supplemented with pterostilbene at a 30 mg/kg body weight/d dose (PT30 group) or a high-fat high-fructose diet supplemented with resveratrol at a 30 mg/kg body weight/d dose (RSV30 group) for eight weeks**.** Values are presented as mean ± SEM. Bars not sharing a common letter are significantly different (*p* < 0.05). CPT-1A: carnitine palmitoyltransferase-1A; CS: citrate synthase; NRF1: nuclear respiratory factor 1; SIRT3: sirtuin 3

**Fig. 3:**
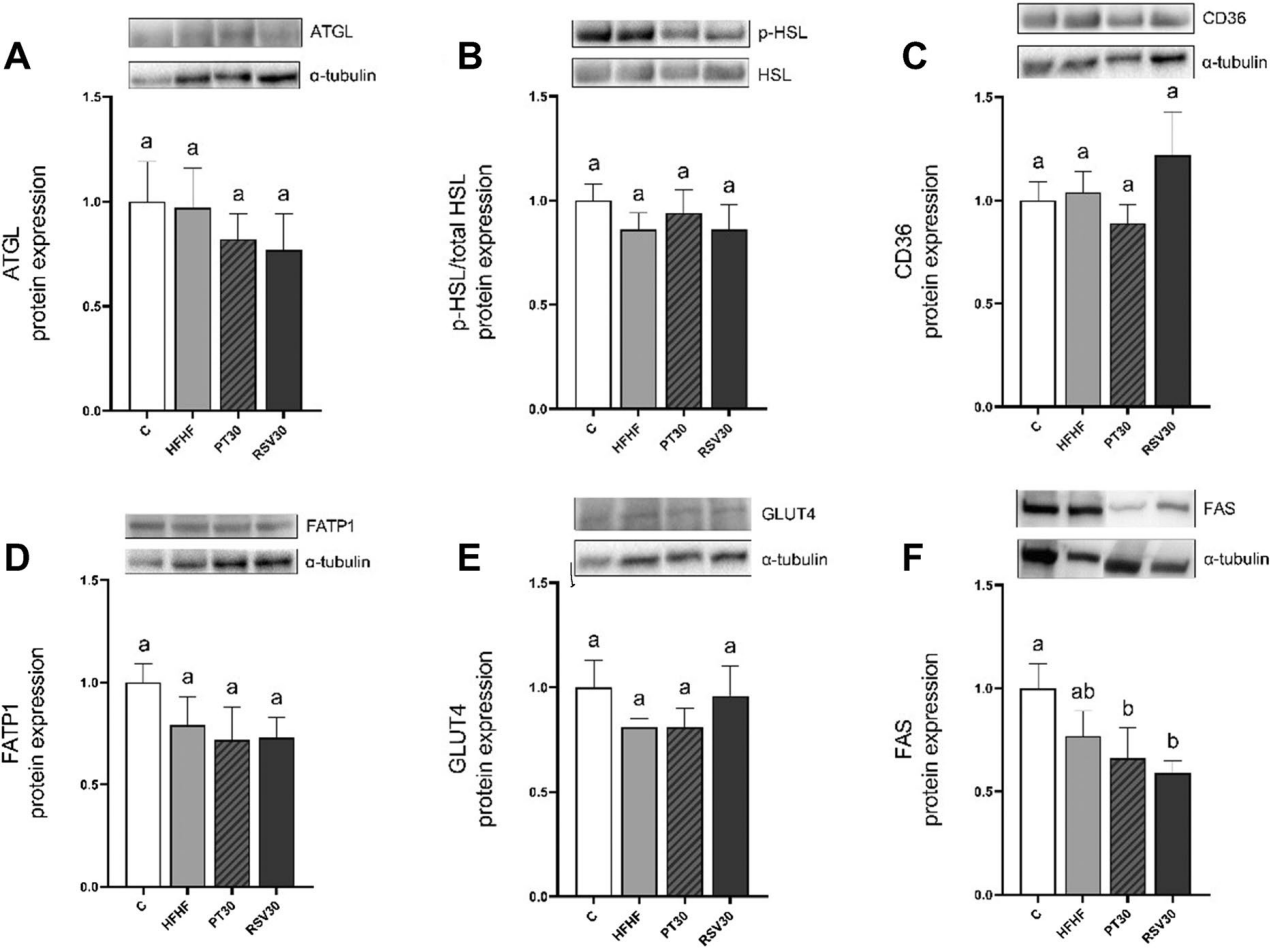
Protein expression of ATGL (**A**), p-HSL/HSL (**B**), CD36 (**C**), FATP1 (**D**), GLUT4 (**E**) and FAS (**F**) in iBAT from rats fed with a control diet (C group), a high-fat high-fructose diet (HFHF group), a high-fat high-fructose diet supplemented with pterostilbene at a 30 mg/kg body weight/d dose (PT30 group) or a high-fat high-fructose diet supplemented with resveratrol at a 30 mg/kg body weight/d dose (RSV30 group) for eight weeks. Values are presented as mean ± SEM. Bars not sharing a common letter are significantly different (*p* < 0.05). ATGL: adipose triglyceride lipase; CD36: cluster of differentiation 36; FAS: fatty acid synthase; FATP1: fatty acid transport protein 1; GLUT4: glucose transporter 4; HSL: hormone-sensitive lipase; p-HSL: phosphorylated hormone-sensitive lipase

Moreover, CPT-1A activity was assessed, and significantly greater enzyme activity was observed in HFHF compared to the C group (*p* < 0.001), while this effect was completely prevented in both PT30 and RSV30 groups (Fig. 2A). With regard to lipolysis, protein expression of ATGL and HSL were measured, yielding no differences among groups in both ATGL and HSL protein expression levels (Fig. 3A and B). In addition, the protein expression of CD36 and FATP1 fatty acid transporters in brown adipocytes were analysed. In this regard, no changes were observed among the experiential groups (Fig. 3C and D).

Due to the fact that mitochondrial activity is a process closely related to thermogenesis, several proteins, such as NRF1, SIRT3 and CS, were analysed. Regarding NRF1, in comparison with the control group, a tendency towards higher levels in HFHF was observed (+ 70%; *p* = 0.08). Moreover, the PT30 group completely prevented this effect, while RSV30 did not avoid the influence of the HFHF diet (Fig. 2B). Regarding CS, though no changes were observed between C and HFHF groups, a tendency towards lower protein activity was found in the PT30 group when compared with rats fed with an HFHF diet (-17.4%; *p* = 0.07) (Fig. 3C). Finally, as far as SIRT3 is concerned, the group supplemented with RSV30 showed a significant increase in protein expression levels compared to the HFHF group (*p* < 0.05), while this effect was not seen in rats treated with pterostilbene (PT30). Lastly, no changes in SIRT3 expression levels were observed between HFHF and C groups (Fig. 2D).

In order to analyse potential differences in glucose uptake by brown adipocytes for de novo lipogenesis, GLUT4 transporter was studied; however, no significant changes were observed among the experimental groups (Fig. 3E). Concerning the lipogenic enzyme FAS, a decrease in FAS protein expression with a large effect size (Cohen’s *d* = -1.9) was observed in HFHF group compared to C group. Likewise, both PT30 and RSV30 prompted a significant decrease in their protein expression compared to the C group (*p* < 0.05 and *p* < 0.01 respectively). Moreover, both PT30 and RSV30 showed a tendency towards lower levels than the HFHF group (-28.3%; *p* = 0.09 and -23.2%; *p* = 0.09, respectively), with a large effect size of the treatment (Cohen’s *d* = -1.8% and -2%, respectively) (Fig. 3F).

**Fig. 4:**
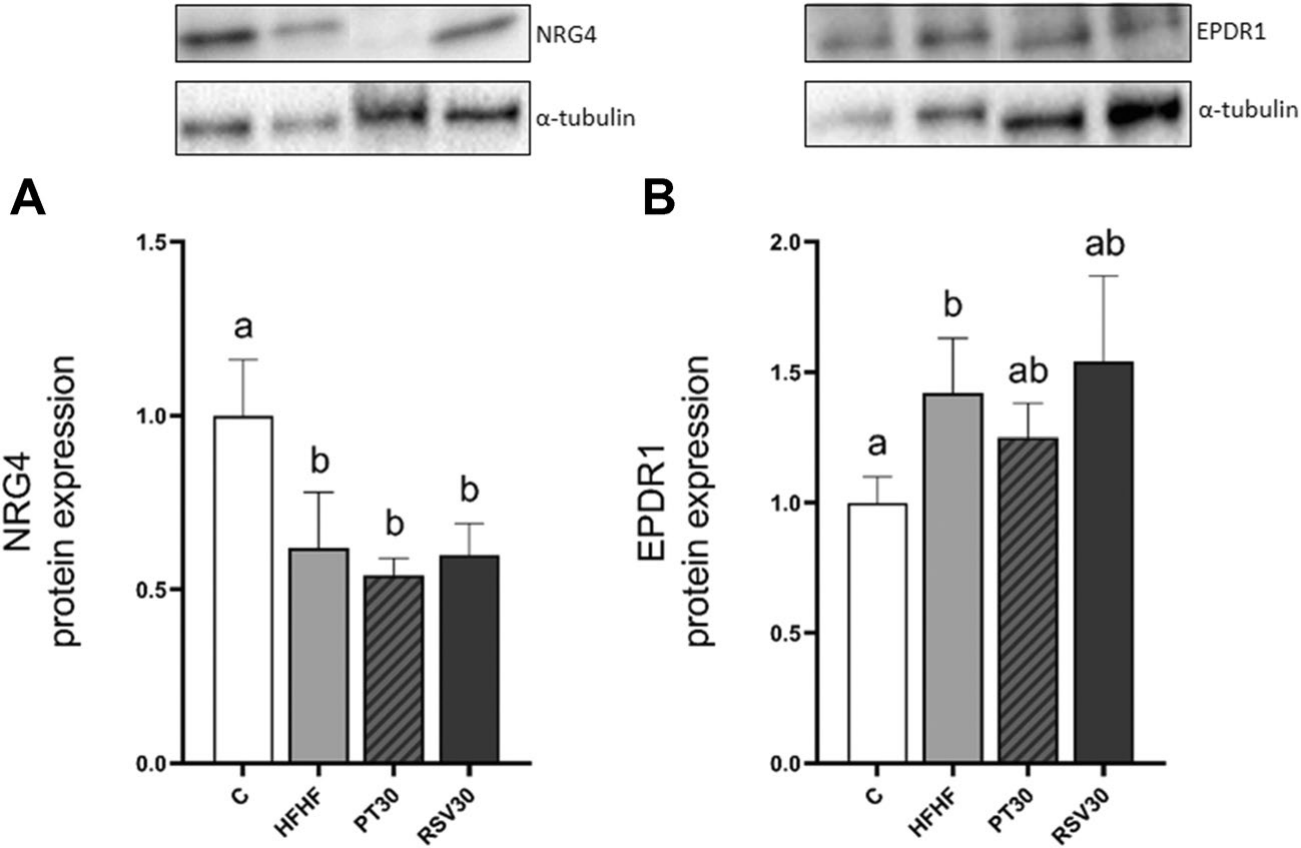
Protein expression of NRG4 (**A**) and EPDR1 (**B**) in iBAT from rats fed with a control diet (C group), a high-fat high-fructose diet (HFHF group), a high-fat high-fructose diet supplemented with pterostilbene at a 30 mg/kg body weight/d dose (PT30 group) or a high-fat high-fructose diet supplemented with resveratrol at a 30 mg/kg body weight/d dose (RSV30 group) for eight weeks. Values are presented as mean ± SEM. Bars not sharing a common letter are significantly different (*p* < 0.05). EPDR1: ependymin related 1; NRG4: neuregulin 4

Finally, taking into account the possible role that batokines play in BAT metabolism, the protein expression of some of them was measured. NRG4, which is related to BAT activation [6], and EPDR1, which plays an important role in BAT adipogenesis [12]. Thus, HFHF showed a significant decrease in NRG4 protein expression levels compared to the C group (*p* < 0.05). However, in the case of the phenolic compound supplementation, neither PT30 nor RSV30 was able to prevent this effect, which is induced by the HFHF diet (Fig. 4A). Greater protein levels of EPDR1 were found in the HFHF group than in the rats fed with the control diet (*p* < 0.05) (Fig. 4B). Finally, compared to the HFHF group, no significant change in EPDR1 expression was found in rats supplemented with neither PT30 nor RSV30.

## Discussion

Resveratrol administration has been shown to increase thermogenic capacity in rodent models [15, 33, 37]. Nevertheless, due to its low bioavailability [50, 52], its dimethoxy derivative pterostilbene has created great expectations as a result of its higher resistance to phase II metabolism. This compound presents one single hydroxyl group, which increases its transport into cells, as well as its metabolic stability [21]. In fact, in our research group it has been reported that, when the same dose of pterostilbene and resveratrol was administered to the rats, the concentrations of the parent compound and their metabolites in the liver were higher in the case of pterostilbene. In addition to that, in the case of pterostilbene the most abundant metabolite was pterostilbene-4′-sulfate, whereas in the case of resveratrol was resveratrol-3-glucuronide [18]. It is noteworthy that scarce information has been reported so far concerning the effects of pterostilbene on thermogenic capacity [1, 24]. Therefore, to the best of our knowledge, this is the first time that the effect of both phenolic compounds on BAT metabolism has been analysed, under the same experimental conditions, in order to make a solid comparison.

In the present study, rats were fed a diet rich in saturated fat and fructose for eight weeks. This dietary pattern induced liver steatosis, but surprisingly not obesity. In fact, this dietary intervention prompted a great increase in liver fat, since 78% of rats under the HFHF diet showed grade 2 liver steatosis [18]. However, obesity was not developed because, although final body weight significantly increased, no changes were observed in the size of different adipose tissue depots and the adipose index. While the vast majority of the studies reported have analysed the effects of stilbenes in models of obesity, we considered it would be interesting to assess the effects of these compounds on brown adipose tissue in this steatotic and non-obese rat model, which had been maintained under high-fat high-fructose feeding conditions, to better characterise their potential thermogenic action.

In the HFHF model, iBAT triglyceride accumulation was increased, although this boost was not high enough to significantly enhance the tissue weight. Different lipid metabolic pathways were explored by measuring protein expressions or activities. Thus, two key proteins implicated in fatty acid oxidation and mitochondriogenesis, CPT-1a and NRF1, respectively, were up-regulated in this group of rats, although in the case of NFR-1, only a trend was found. These results are in good accordance with those found after analysing the liver of these rats; where CPT-1a as well as mitochondriogenesis-related proteins, were also increased, suggesting a greater fatty acid oxidation rate [18]. This change can represent a compensatory mechanism devoted to avoiding excessive triglyceride accumulation in the tissue. By contrast, de novo lipogenesis (FAS), lipolysis (ATGL, HSL) and fatty acid uptake (CD36, FATP1) were not modified by HFHF feeding. Interestingly, a trend towards higher values of UCP1 protein expression was observed. This result is in good accordance with the increase in triglyceride content found in IBAT. In fact, it has been reported that the enhancement in iBAT triglycerides, resulting from high-fat feeding, is accompanied by increased UCP1 values [11, 23, 36]. All in all, these results suggest that the rise in triglyceride content observed in iBAT can be due to the increased availability of fatty acids coming from circulating triglycerides, as a consequence of the high amount of fat in the diet. It can also be inferred that, in relation to the lack of GLUT-4 transporter protein expression, fatty acids, albeit not glucose, could be the substrate source for a potential increase in thermogenesis.

Concerning the effects of the phenolic compounds (resveratrol and pterostilbene at 30 mg/kg/day) at the end of the eight weeks, interestingly, they fully prevented the increase in iBAT triglyceride accumulation, where the values exhibited by the rats in PT30 and RSV30 groups did not differ significantly from the control animals. In these groups, the lipid metabolic pathways that had not been affected by HFHF feeding remained unaltered. By contrast, the boost induced in CPT-1 activity was fully prevented, thus, giving support to our hypothesis that the increase observed in HFHF groups was a compensatory mechanism. Indeed, this mechanism was no longer necessary in the rats supplemented with pterostilbene or resveratrol. In the case of the PT30 group, the phenolic compound supplementation also avoided the increase induced by HFHF in NFR1 protein expression.

Regarding the effects on UCP1, the tendency towards higher values of UCP1 observed in pterostilbene-treated rats fits well with the propensity to lower values of the acetylated PGC-1α/total PGC-1α ratio, which indicates activation of this transcription co-factor. Actually, it is known that PGC-1α activation leads to up-regulation of UCP1 [30, 48]. For an effective activation of thermogenesis, the up-regulation of UCP1 should be accompanied by grows in mitochondria number or activity, devoted to enhancing fatty acid oxidation [20, 26, 49]. However, this effect was not induced in the PT30 group. Altogether, these results suggest that, although changes targeted to increasing thermogenesis were induced, pterostilbene treatment did not activate the integral thermogenic machinery, and thus, increased thermogenesis is not expectable in the PT30 group.

In the case of resveratrol-treated rats, a significant raise in both UCP1 and SIRT3 was observed. SIRT3 is a master regulator of mitochondrial metabolism, which is activated during thermogenesis [22, 42]. In fact, this sirtuin appears to influence oxidative metabolism by controlling mitochondrial biogenesis [17]. However, no significant changes in PGC-1α, the other master regulator of mitochondriogenesis, were found. Moreover, as in the case of pterostilbene, elevated mitochondria number or activity were not induced, and consequently, increased thermogenesis was not likely.

When comparing the effects of resveratrol and pterostilbene observed in this study with those found in animal models of obesity, important differences can be descried. In this line, in a study conducted by our group and carried out in genetically obese Zucker rats treated with 15 or 30 mg/kg/d of the phenolic compound for six weeks, increased gene expressions of *ucp1, pparα, nfr-1* and *cox-2,* as along with increased protein expression of UCP1, PPARα and higher enzymatic activities of CPT-1a and CS were observed in brown adipose tissue, meaning that pterostilbene augmented thermogenic and oxidative capacities of this tissue [1]. Similarly, Wistar rats rendered obese by high-fat high-sucrose feeding, and further supplemented with resveratrol for six weeks, showed significantly raised UCP1, SIRT3 and TFAM protein expression, as well as activation of AMPK and enhanced CPT-1a and CS activities [34]. Altogether, these data suggest that the effects of stilbenes on brown adipose tissue thermogenic capacity depend on the metabolic status, and more precisely on the presence or absence of obesity, although further studies are needed to confirm this hypothesis.

As previously mentioned, iBAT displays an inability to produce batokines. These molecules can act as lipid metabolism regulators in their own tissue producer and also in other tissues and organs, such as the liver [2, 16, 27, 32, 44]. In the present study, NRG4 and EPDR1 were analysed. It is well known that NRG4, secreted in higher amounts by iBAT, inhibits hepatic de novo lipogenesis and expression of genes involved in this process, thus, establishing a crosstalk iBAT-liver that attenuates hepatic steatosis [38]. After HFHF feeding, a significant down-regulation of NRG4 was observed, suggesting that the suppressive effect of NRG4 on hepatic de novo lipogenesis was reduced in rats under this feeding pattern. Indeed, this was the actual situation in this cohort of rats, as previously reported by our group, because the activity of FAS was significantly higher in the HFHF group than in the control group [18]. This result is in good accordance with that reported by Zhu B. et al*.* [54] where NRG4 levels were attenuated in aged mice fed with a high-fat diet. Regarding phenolic compound supplementation, neither PT30 nor RSV30 was able to restore NRG4 levels to those found in rats under HFHF feeding. As far as EPDR1 is concerned, it has been reported that it activates the adipogenic pathway in brown adipose tissue [12]. In the present study, the HFHF diet intervention increased EPDR1 levels in iBAT, suggesting greater adipogenesis in this tissue, which is related to the increase in IBAT triglyceride content observed in the experimental group. No changes were induced by the phenolic compounds.

This study has some limitations. On the one hand, no functional measurements of thermogenesis such as tissue oxygen consumption were addressed, and thus, only considerations about thermogenic capacity can be ruled out. On the other hand, due to the limited size of iBAT, the activation of some proteins was not measured.

## Conclusions

The results obtained show that, although some key proteins related to thermogenesis were modified by either pterostilbene or resveratrol, the lack of impact on other important proteins of the thermogenic machinery suggests that these compounds were not able to stimulate thermogenesis in iBAT under the present experimental conditions, which is in sharp contrast with their effects on obesity models. In addition, the alterations in some batokine secretion induced by the HFHF diet were not prevented by these phenolic compounds.

## Data Availability

The authors confirm that the data supporting the findings of this study are available within the article.
